# The Central Entry Point for Infra‐Acetabular Screw Placement: Defining an Anatomical Entry Zone and Evaluating Its Clinical Efficacy

**DOI:** 10.1111/os.70384

**Published:** 2026-07-24

**Authors:** Jiaqiang Chen, Yongsheng Wang, Peishuai Zhao, Renjie Li, Xiaopan Wang, Xiaotian Chen, Jianzhong Guan, Min Wu

**Affiliations:** ^1^ Department of Orthopaedics The First Affiliated Hospital of Bengbu Medical College Bengbu China

**Keywords:** acetabular fracture, entry point, infra‐acetabular screw

## Abstract

**Objective:**

Previous studies have been constrained by a single ideal entry point and a narrow bony corridor, leaving a limited selection of entry points for infra‐acetabular screws and resulting in suboptimal surgical efficiency. This study aims to analyze the morphology of the infra‐acetabular screw entry zone and evaluate a central entry point zone for optimizing surgical screw placement.

**Methods:**

3D pelvic models from 100 patients were reconstructed. The entry zone was morphologically analyzed, and its similarity to a standard ellipse was calculated. A clinical comparison was conducted on 20 patients with acetabular fractures, comparing the central entry point technique against the ideal entry point method.

**Results:**

The entry zone exhibited a quasi‐elliptical shape. The central entry point was consistently located medial and anterior to the ideal entry point. Clinically, the central entry point group demonstrated significantly shorter operative time and fewer x‐ray exposures, while screw length and success rate showed no significant difference.

**Conclusion:**

The infra‐acetabular screw entry zone is an elliptical structure. Using the central entry point significantly improves placement efficiency and reduces radiation exposure, offering considerable clinical value.

## Graphical abstract

This study defines the quasi‐elliptical infra‐acetabular screw entry zone, with its center located medial‐anterior to the iliopubic eminence. Utilizing this central entry point significantly improves surgical efficiency, reducing operation time and radiation exposure, while maintaining excellent fixation stability.

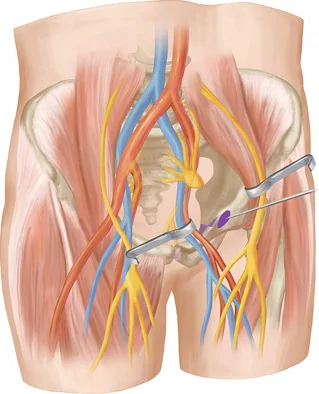

**Abbreviations**
IACD: infra‐acetabular corridor diameter
IPE: iliopubic eminence
LCE Angle: lateral center‐edge angle
MTMAW: minimum thickness of medial acetabular wall
SSIM: structural similarity

## 1 Introduction

Acetabular fractures are common fractures in orthopedics, with an incidence rate of 3 per 100,000, a mortality rate of 5%–20%, and a disability rate of 50%–60% [1, 2]. Unlike the trend toward minimally invasive treatment for simple pelvic fractures, acetabular fractures are intra‐articular fractures located deep within the anatomy and typically require open reduction. The acetabulum is adjacent to numerous vital organs and has a complex anatomy, making fracture reduction and fixation challenging [3]. Periacetabular lag screws combined with plates provide excellent fixation of the acetabulum, enabling patients to initiate early rehabilitation exercises and reducing the incidence of complications. The infra‐acetabular screw is a crucial component of the acetabular lag screw system. The infra‐acetabular screw was first proposed by Culemann in 2011. A narrow trabecular bone corridor exists along the medial border of the iliopubic eminence (IPE), running parallel to the posterior border of the obturator foramen and directed toward the ischial tuberosity. This screw fixation is indicated for treating anterior–posterior column separation fractures (anterior column fractures, T‐shaped fractures, fractures of the anterior column or anterior wall with posterior hemi‐transverse fracture, and fractures of both columns) [4, 5]. Infra‐acetabular screws provide excellent fixation, enhancing the stability of plate‐screw systems by up to 50% [6]. This configuration has been shown to achieve the specified strength required for 3D steel plates [7]. Wang [8] also demonstrated through acetabular posterior column pelvic models that the additional placement of infra‐acetabular screws significantly enhances the fixation efficacy of posterior column screws for posterior column fractures. Infra‐acetabular screw fixation offers excellent results and broad applicability, but the narrow corridor requires at least a 5 mm bony channel for insertion of a 3.5 mm screw [9]. Therefore, anatomical research on the infra‐acetabular corridor is essential.

Based on an analysis of 523 pelvic CT scans, Gras found that 94% of males and 90% of females possess an infra‐acetabular corridor [10]. Lu [11] found that in an Asian study population, only 76% of men and 66% of women were suitable candidates for infra‐acetabular screw placement. The narrow screw corridor demands precise entry points and direction. This places high demands on the surgeon's experience and screw placement technique [12]. Culemann [4] proposed that the ideal entry point for the infra‐acetabular screw is 1 cm caudal to the IPE. Baumann [13] calculated through CT image analysis that the average entry point for an ideal infra‐acetabular screw is located 10.2 mm caudally and 10.4 mm medially from the IPE. However, during clinical surgery, due to the curvature of the pelvic arch and the coverage of fracture lines, the plate holes are not always precisely located at the ideal entry point. Inserting the screw far from the ideal entry point can easily cause the corridor to deviate, leading to an “in‐out‐in” phenomenon and resulting in iatrogenic injury. Current literature on infra‐acetabular screws is largely limited to studies on entry point and screw direction. This study focuses on reconstructing the entry zone for infra‐acetabular screws, thereby expanding the range of insertion options for these screws. This study conducted a retrospective analysis, randomly selecting 100 patients (50 males, 50 females) who underwent pelvic CT thin‐slice scanning at our institution. The pelvic models were extracted using Mimics software (version 21.0; Materialise NV, Leuven, Belgium), and the placement of infra‐acetabular screws was simulated using 3‐matic software (version 13.0; Materialise NV, Leuven, Belgium). Adjust the transparency of the 3D reconstruction model to enable axial visualization of the infra‐acetabular screws. Determine the ideal entry point, length, and corridor diameter for infra‐acetabular screws. Adjust the orientation and position of the infra‐acetabular screw, explore all permissible entry zones, and analyze their morphology. Based on the morphological parameters of the entry zone, 20 patients with anterior–posterior column separation fractures were ultimately included for clinical application. Based on the above, the purposes of this study were: (i) to determine the ideal entry point, length, and bony corridor diameter of the infra‐acetabular screw through 3D simulation analysis, while comprehensively exploring all permissible entry zones; (ii) to reconstruct and characterize the morphology of the entry zone, moving beyond a single ideal entry point to expand the range of screw placement options; and (iii) to apply the obtained morphological parameters to clinical practice involving 20 patients with associated anterior and posterior column fractures, thereby validating the clinical feasibility of this approach.

## 2 Materials and Methods

### 2.1 Data Collection

A retrospective systematic sampling study included the CT data of 100 patients who underwent thin‐slice CT scans of the pelvis between December 2023 and December 2024, which were used to reconstruct 3D models. Among them, 50 were male and 50 were female, with an average age of (49.72 ± 11.87) years (range: 20–68 years). Inclusion Criteria: (1) Inpatients who underwent CT scanning at our institution; (2) Age 20–70 years. Exclusion Criteria: (1) Abnormal pelvic structure caused by congenital developmental or acquired diseases; (2) Height greater than 1.9 m or less than 1.5 m (This height range was selected based on previous anatomical studies on pelvic morphology in Asian populations to avoid confounding effects from extreme pelvic size differences on bone channel measurements [11, 14]); (3) Cases in which neither entry point is accessible for screw simulation. (4) Individuals with a history of pelvic tumors or prior pelvic surgery.

In addition, 20 patients admitted to our institution between January and June 2025 were selected for clinical validation and comparison. Inclusion criteria: (1) Age ≥ 18 years; (2) Acetabular fractures classified as anterior column fractures, T‐type fractures, anterior column with posterior transverse fractures, or fractures of both columns according to the Letournel‐Judet classification; (3) Patients undergoing surgical treatment with an infra‐acetabular screw placed at the central entry point during surgery. Exclusion criteria: (1) Time from fracture to surgery > 3 weeks; (2) History of osteoarthritis or total hip arthroplasty in the ipsilateral hip.

**FIGURE 1:**
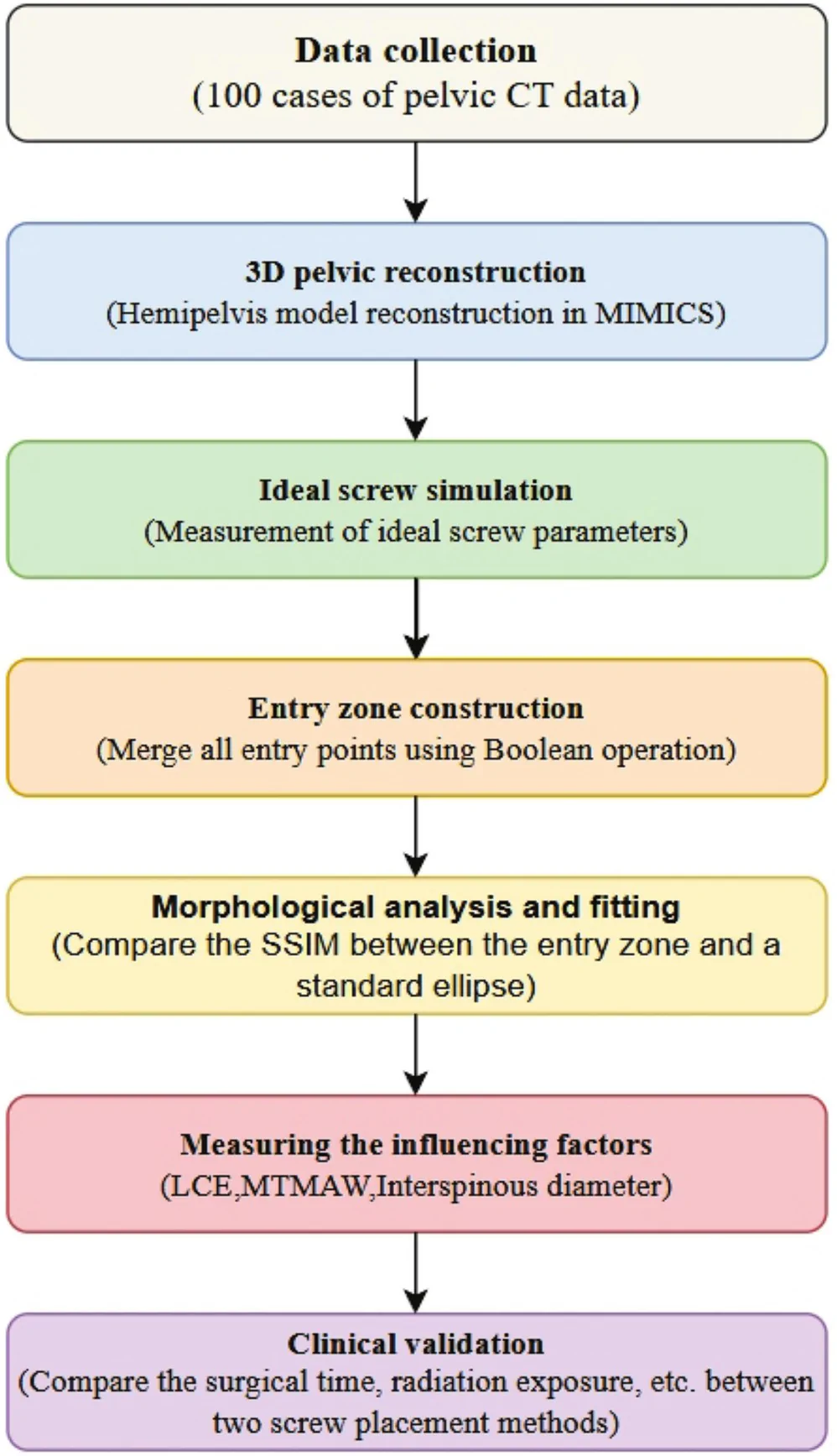
3D modeling and morphological analysis workflow of the infra‐acetabular screw. LCE, lateral center‐edge angle; MTMAW, minimum thickness of medial acetabular wall; SSIM, structural similarity.

The workflow of 3D modeling and analysis is as follows (Figure 1). This study adheres to the guidelines of the Declaration of Helsinki and was approved by the Medical Ethics Committee of our institution (Approval No. 2023YJS180), and all patients consented to the use of their anonymized CT data for research purposes. All patients provided written informed consent.

### 2.2 Establishment of the Pelvic Model

Import CT scan data in DICOM format into Mimics 21.0. Select bone tissue by adjusting threshold values (226 HU–3071 HU). Use editing tools and segmentation masks within the virtual model to reconstruct the pelvic structure. Use the Smart Fill tool to select unselected bone edges and fill hollow bone structures. Analyze the right hip bone; if fractures or deformities occur on the right side, use the Symmetry tool to adjust the left hip bone to match the right. The anatomical accuracy of the symmetry‐adjusted model was validated by matching key bony landmarks (iliopubic eminence, ischial tuberosity) and cortical bone pixel values of the left and right hemipelvises.

### 2.3 Ideal Screw Parameter Measurement

**FIGURE 2:**
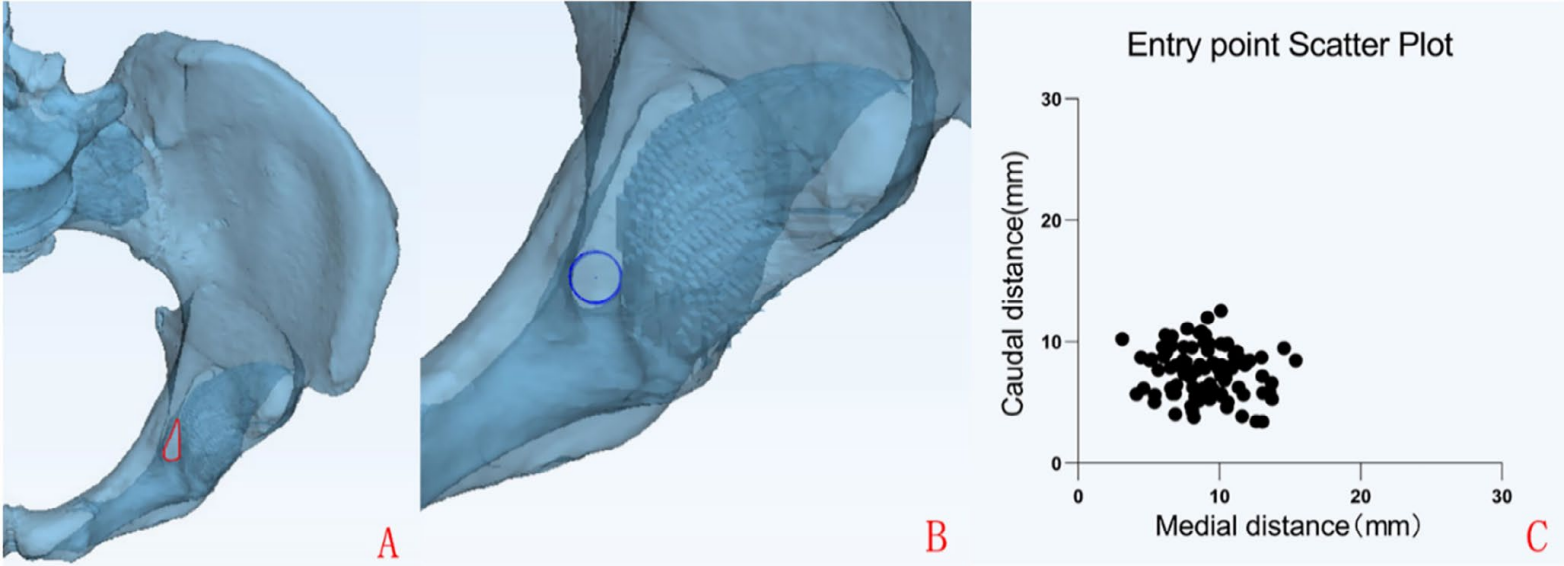
(A) Infra‐acetabular screw corridor perspective view; (B) Ideal infra‐acetabular screw diagram; (C) Ideal entry point scatter plot.

Import the hip bone model into three‐matic 13.0. Increase the model's transparency using the Transparency command. The screw insertion corridor for the infra‐acetabular screw can then be visualized using axial perspective (Figure 2A). Insert the 3.5 mm cylindrical simulated screw into the model, reduce its transparency, and rotate the pelvic model to ensure the simulated screw does not protrude beyond the bone. Gradually increase the screw diameter until it cuts through the bone. Adjust the transparency of the bone, rotate, and scale the 3D view to visually assess the screw position and confirm no cortical breach. Then, corroborate the visual assessment with CT slices (axial/coronal/sagittal views). The diameter of this screw corresponds to the IACD (Infra‐acetabular Corridor Diameter). Record IACD (Figure 2B). Set the cylinder radius to 1 mm. Record the point where the screw contacts the cortical bone as the ideal entry point. Record the screw length as the ideal screw length. Centered on the IPE apex, measure the horizontal and vertical distances of the ideal entry point relative to the IPE apex, denoted as X and Y. Record these values in a Cartesian coordinate system and plot them as a scatter diagram (Figure 2C).

### 2.4 Entry Zone Surface Area Measurement

**FIGURE 3:**
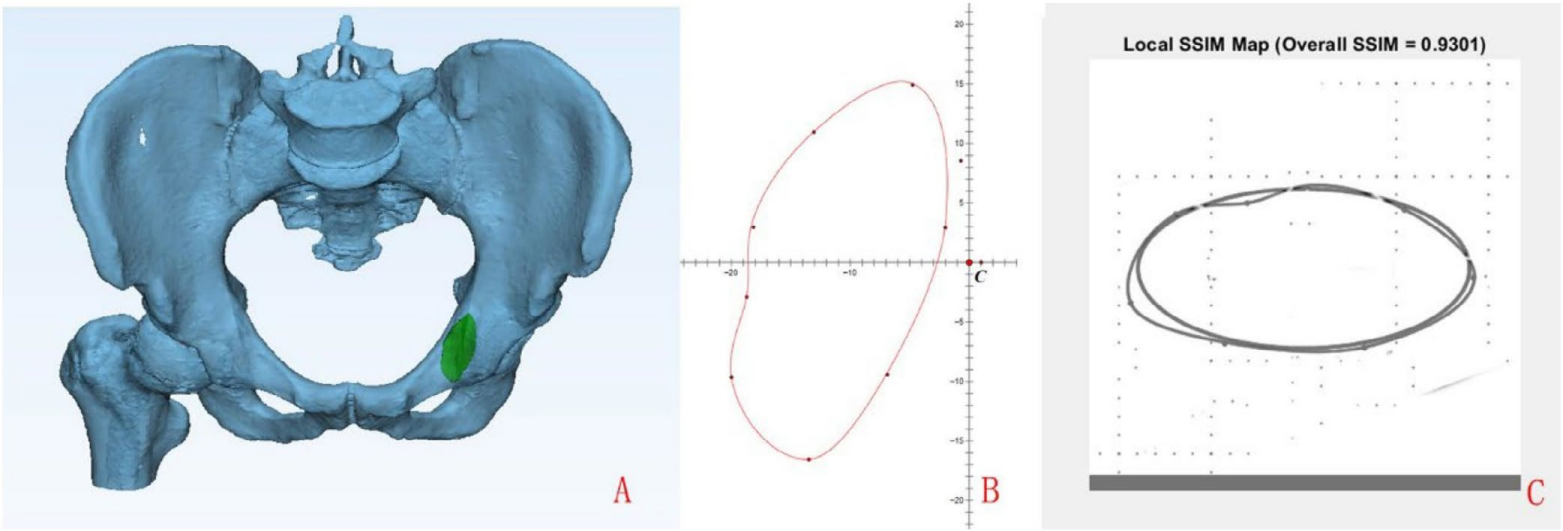
(A) Infra‐acetabular screw entry zone diagram; (B) coordinates of each infra‐acetabular screw entry point; (C) comparison of entry zone similarity with ideal elliptical structure.

After simulating an ideal screw in 3‐matic 13.0, repeatedly adjust the model position around this center point and place new simulated screws with a diameter of 3.5 mm. Continuously adjust transparency and rotate the view during screw placement to ensure the screw remains within the bone structure at all times, simulating screw placement until all bone corridors are occupied. By performing a Boolean operation to merge all screws, the intersection between the screws and the pelvis forms the 3D shape of the infra‐acetabular screw corridor. The region where the intersecting surfaces coincide with the upper bony surface constitutes the entry zone. By using the Surface‐Freeform Patch command to smoothly connect each entry point, the entry zone S for the infra‐acetabular screw can be obtained (Figure 3A). The entry zone is roughly elliptical. Record the coordinates of the endpoints of the two major axes, the endpoints of the minor axis, and the midpoints of the four endpoints. Using the apex of the iliopubic eminence as the coordinate center, denote the horizontal and vertical distances of each coordinate point relative to the apex as “x and y,” respectively. Connect these points with a smooth curve (Figure 3B).

### 2.5 Measurement of Morphological Parameters and Morphological Matching of the Entry Zone

After connecting each entry point with a smooth curve in the coordinate system, the entry zone is observed to be approximately elliptical. Connect the endpoint of the major axis with the endpoint of the minor axis. The coordinates of the intersection point “O” of the major and minor axes are denoted as (x, y). The radii of the major axis are denoted as R_1_ and R_1_′, with their average denoted as ^−^R_1_ The radii of the minor axis are denoted as R_2_ and R_2_′, with their average denoted as ^−^R_2_. Establish a standard ellipse using ^−^R_1_ and ^−^R_2_ as its two radii. Import both image files into MATLAB 2024, convert them to grayscale, and force resize them to match dimensions. Compare their structural similarity (SSIM) using the SSIM function from the image processing toolbox (Figure 3C).

### 2.6 Entry Zone Influencing Factor Measurement

**FIGURE 4:**
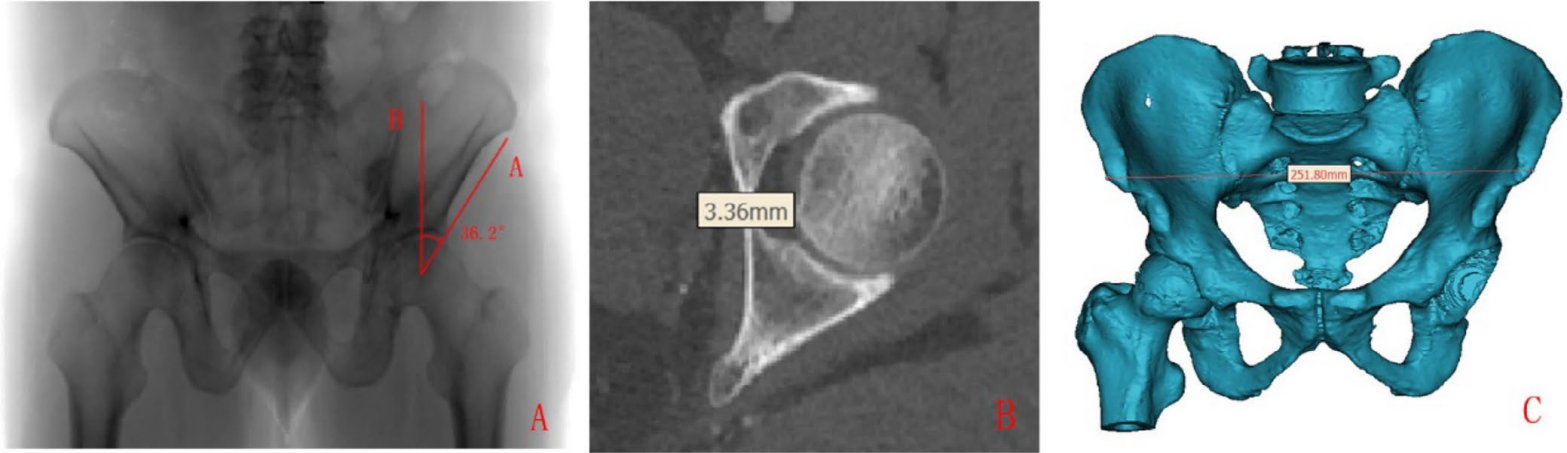
(A) Lateral center‐edge angle measurement diagram; (B) minimum thickness of medial acetabular wall measurement diagram; (C) interspinous diameter measurement diagram.

After importing CT scan data into Mimics 21.0, generate simulated x‐ray images using the “Create DRR” tool under “Tools.” Set the source‐image distance (SID) to 100 cm, the projection direction to anteroposterior (AP), the simulated x‐ray energy to 100 kVp, and the aluminum filter. Adjust the window width and level to encompass the pelvis, then optimize contrast and brightness to obtain the simulated x‐ray image. Draw a straight line (Line A) from the center of the femoral head to the lateral margin of the acetabulum. Draw a perpendicular line (Line B) to the horizontal midline of the pelvis (the line connecting both ischial tuberosities). The angle α between lines A and B represents the LCE (Lateral Center‐Edge Angle) (Figure 4A). In Mimics 21.0, using the interactive MPR tool, rotate the indicator parallel to the bilateral anterior superior iliac spines to reconstruct the standard cross‐section. Set the slice thickness to 0.625 mm. Measure the thickness of the medial acetabular wall on each slice and record the minimum value as Minimum Thickness of Medial Acetabular Wall (MTMAW) (Figure 4B). In the pelvic reconstruction model, align the model in the anteroposterior position. Measure the distance between the outer edges of the bilateral anterior superior iliac spines to obtain the interspinal diameter, denoted as d (Figure 4C).

### 2.7 Clinical Case Inclusion

**FIGURE 5:**
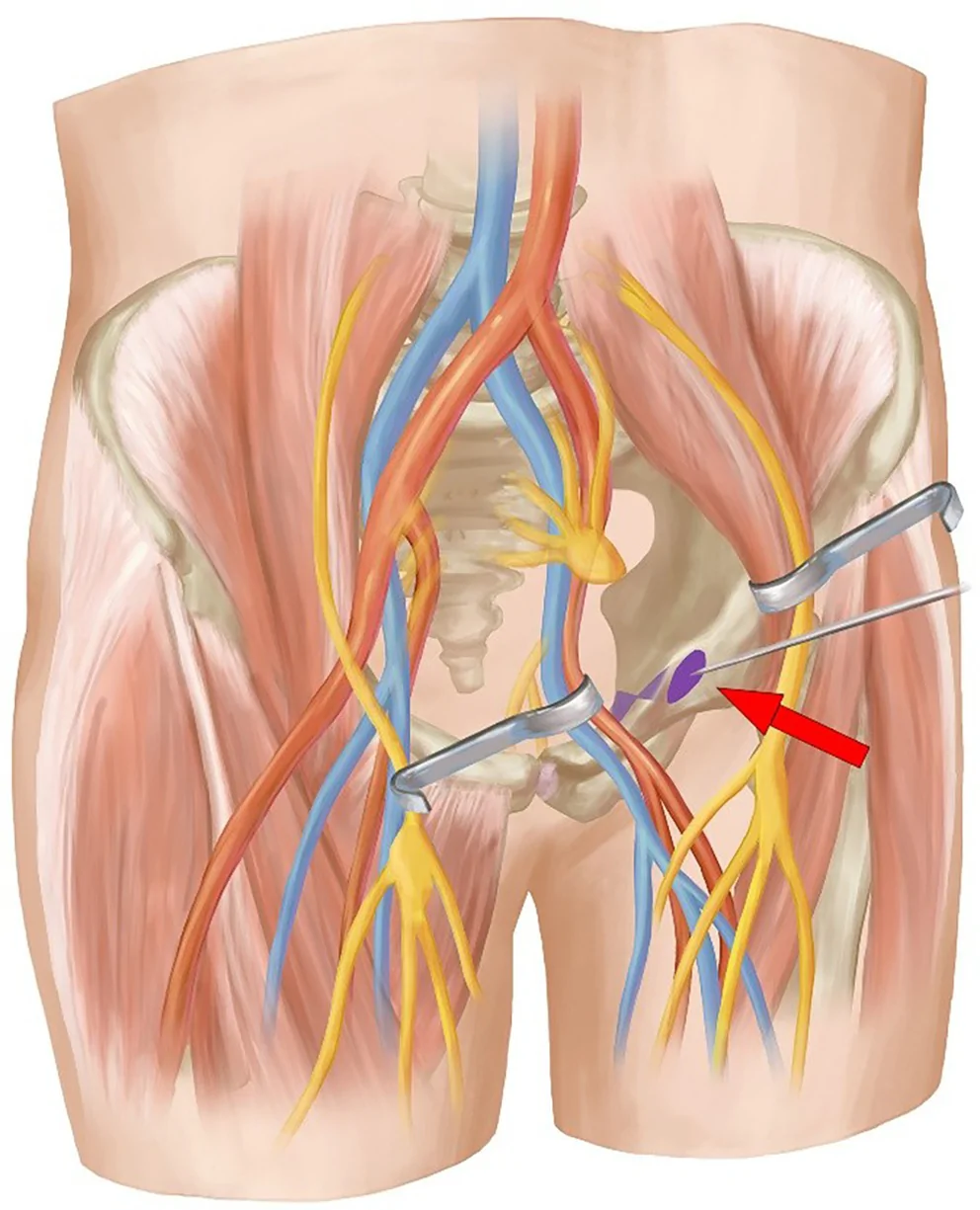
The purple area where the guide screw contacts the bone is the entry zone. The surgeon can freely choose the insertion point for the infra‐acetabular screw within this area.

The experimental group consisted of 20 patients in whom the infra‐acetabular screw was placed according to the central entry point and entry zone (Figure 5), and the control group consisted of 34 patients in whom the screw was placed according to the ideal entry point. The baseline characteristics were compared between the two groups to exclude confounding factors. The details of the corresponding tests can be found in the Statistical Methods section. The following parameters were recorded: screw length, insertion time, number of exposures, and screw placement success rate.

### 2.8 Statistical Analysis

Statistical analysis was performed using SPSS 26.0 (IBM Corp., Armonk, NY, USA). For continuous variables, the Shapiro–Wilk test was first used to assess normality, *p* > 0.05 indicating a normal distribution. The following variables were found to be normally distributed with homogeneous variances: ideal screw length, ideal screw diameter, entry area, ideal entry point parameters, central entry point parameters, R1, R1′, R2, R2′, SSIM, LCE angle, MTMAW, interspinous distance d, actual screw length, screw placement time, and fluoroscopy exposure count. These variables are presented as mean ± standard deviation (x̄ ± s). Comparisons between male and female patients were performed using the independent‐samples *t*‐test. Within‐subject comparisons between long‐radius and short‐radius parameters (e.g., R1 vs. R1′, R2 vs. R2′) were performed using the paired *t*‐test. Age was not normally distributed and is presented as median (interquartile range); between‐group comparisons for age were performed using the Mann–Whitney U test. Categorical variables (e.g., sex, fracture type, treatment outcome) are presented as frequencies and percentages. Sex composition was compared using the chi‐square test. Between‐group comparisons of fracture type and treatment outcome were performed using Fisher's exact test. The significance level was set at α = 0.05 (two‐tailed), and *p* < 0.05 was considered statistically significant.

## 3 Results

### 3.1 Parameters of the Ideal Infra‐Acetabular Screw

**FIGURE 6:**
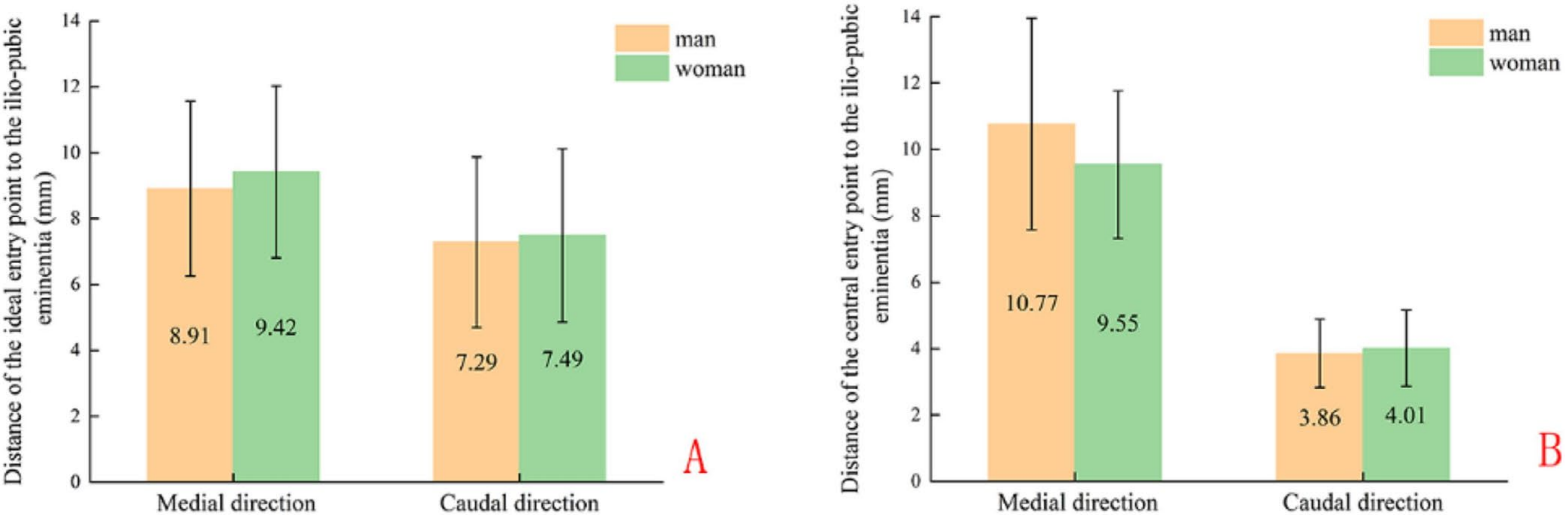
The distance from the ideal entry point and the central entry point to the IPE.

Seventy‐eight percentage of males (39/50) and 72% of females (34/50) were suitable for placement of an infra‐acetabular screw (χ^2^ = 1.2684, *p* > 0.05), with no statistically significant difference. The ideal entry points for infra‐acetabular screws in males and females were: medial IPE (9.42 ± 2.61) mm, caudal (7.49 ± 2.63) mm, with no statistically significant difference (*t*_*x*_ = 1.203, *p* = 0.232; *t*_*y*_ = 1.532, *p* = 0.130). (Figure 6A) The lengths of the infra‐acetabular screws in males and females were (104.03 ± 3.97) mm and (88.72 ± 3.72) mm (t = 16.936, *p* < 0.05), respectively. The infra‐acetabular corridor diameters were (6.71 ± 0.96) mm and (5.7 ± 0.76) mm, respectively, with significant differences between males and females (*p* < 0.05).

### 3.2 Entry Zone Parameters and Influencing Factors of the Infra‐Acetabular Screw

The entry zone under the infra‐acetabular screw was (422.92 ± 80.31) mm^2^ for males and (195.54 ± 66.57) mm^2^ for females, respectively, showing a significant difference between genders (*p* < 0.05). The center coordinates of the entry zone were medial to the IPE at (10.77 ± 3.19) mm and caudal to IPE at (3.86 ± 1.03) mm for males; medial to IPE at (9.55 ± 2.22) mm and caudal to IPE at (4.01 ± 1.14) mm for females (Figure 6B). The differences were not statistically significant (*t*_*x*_ = 1.89, *p* = 0.063; *t*_*y*_ = 0.59, *p* = 0.556) (Table 1). For males, R1 and R1′ measured (17.01 ± 3.53) mm and (15.95 ± 2.16) mm, respectively; R2 and R2′ measured (8.13 ± 1.29) mm and (8.28 ± 1.20) mm, respectively. No statistically significant differences were observed. (*t*_R1_ = 1.580, *p* > 0.05; *t*_R2_ = 0.532, *p* > 0.05). The mean values for R1 and R2 were (16.48 ± 3.04) mm and (8.20 ± 1.19) mm, respectively. For females, R1 and R1′ were (12.96 ± 2.74) mm and (15.70 ± 3.18) mm, respectively; R2 and R2′ were (5.41 ± 0.95) mm and (6.27 ± 1.38) mm, respectively. No statistically significant differences were observed (*t*_R1_ = 1.412, *p* > 0.05; *t*_R2_ = 1.034, *p* > 0.05). The mean values of R1 and R2 were (14.08 ± 3.35) mm and (5.84 ± 1.25) mm, respectively. The similarity between the screw insertion region and the standard elliptical structure, measured by SSIM, was (88.40 ± 5.54) %.

**TABLE 1:** Comparison of infra‐acetabular screw corridor parameters between males and females (x ± s).

Sex	*n*	Screw length (mm)	IACD (mm)	Entry zone area (mm^2^)	SSIM (%)	MTMAW (mm)	LCE Angle (°)	Interspinal diameter (mm)
Male	39	104.03 ± 3.97	6.71 ± 0.96	422.92 ± 80.31	89.21 ± 5.76	4.56 ± 1.24	40.71 ± 6.99	256.31 ± 23.38
Female	34	88.72 ± 3.72	5.70 ± 0.76	195.53 ± 66.57	87.47 ± 5.19	3.33 ± 1.03	38.06 ± 9.15	243.73 ± 16.93
*t*	—	16.94	4.91	13.05	1.34	4.57	1.40	2.60
*p*	—	< 0.001	< 0.001	< 0.001	> 0.05	< 0.001	> 0.05	< 0.05

*Note:* There was no significant difference in the distribution of the 5‐mm bony corridor between the sexes (χ^2^ = 1.2684, *p* > 0.05).

Abbreviations: IACD, infra‐acetabular corridor diameter; LCE, lateral center‐edge angle; MTMAW, minimum thickness of medial acetabular wall; SSIM, structural similarity.

The mean MTMAW measurements for males and females were (4.56 ± 1.24) mm and (3.33 ± 1.03) mm, respectively. The mean Interspinal diameter was (256.31 ± 23.38) mm and (243.73 ± 16.93) mm, respectively. These differences were statistically significant (*p* < 0.05). The LCE angles were (40.71 ± 6.99)° and (38.06 ± 9.15) °, respectively, with no statistically significant difference between males and females (*p* > 0.05).

Multivariate linear regression analysis revealed the approximate elliptical radii of the entry zones:

R1 = 13.88–0.0816LCE + 0.02028 L + 0.08709MTMAW.

R2 = 10.18–0.04891LCE‐0.01564 L + 0.73MTMAW.

### 3.3 Clinical Application of the Entry Zone for the Infra‐Acetabular Screw

All surgeries were performed by the same senior surgeon and their fixed treatment team. The mean ages of the control group and the experimental group were 55.3 and 52.1 years, respectively, with no statistically significant difference (U = 274, *p* = 0.237 > 0.05). According to the Letournel‐Judet classification, the fracture types in the control group included: anterior column (*n* = 7), anterior column with posterior hemitransverse (*n* = 8), T‐type (*n* = 2), and both‐column (*n* = 17). In the experimental group, the fracture types were: anterior column (*n* = 5), anterior column with posterior hemitransverse (*n* = 2), T‐type (*n* = 2), and both‐column (*n* = 11). There was no statistically significant difference in the distribution of fracture types between the two groups (*p* = 0.531 > 0.05). For screws inserted at the center entry point and the ideal entry point, the mean lengths were 85.25 ± 13.03 mm and 86.20 ± 12.16 mm, respectively, with success rates of 85.0% and 82.35%. The difference was not statistically significant (*p* > 0.05) (Tables 2, 3). The average procedure times were 16.00 ± 6.22 min and 20.70 ± 8.31 min, respectively, with average exposure counts of 10.88 ± 3.29 and 16.53 ± 4.42, respectively. The differences were statistically significant (The operation time *p* = 0.035 < 0.05; exposure counts *p* < 0.001) (Table 2).

**TABLE 2:** Comparison of clinical application parameters between the experimental group and the control group.

Group	*n*	Screw length (mm)	Operative times (min)	Exposure frequency (times)
Central entry point	20	85.25 ± 13.03	16.00 ± 6.22	10.88 ± 3.29
Ideal entry point	34	86.20 ± 12.16	20.70 ± 8.31	16.53 ± 4.42
*t*	—	0.26	2.19	4.98
*p*	—	> 0.05	< 0.05	< 0.001

**TABLE 3:** Comparison of fracture reduction quality between the experimental group and the control group.

Group	*n*	Modified Matta criteria				*p*
		Excellent	Good	Fair	Poor	
Central entry point	20	7	10	3	0	0.595
Ideal entry point	34	9	19	6	0	

## 4 Discussion

### 4.1 Main Findings of This Study

This study analyzed 100 pelvic CT scans to validate the anatomical parameters of ideal infra‐acetabular screws. While no significant sex‐specific differences were found in the ideal entry points, males exhibited larger infra‐acetabular corridor diameters and longer screw lengths. The entry zone demonstrated a quasi‐elliptical structure in both sexes, with a significantly larger area observed in males. Furthermore, the central entry point maintained a relatively consistent location, and the morphological radii of the zone were correlated with the LCE angle, MTMAW, and interspinal diameter. Clinically, compared with the ideal entry point method, utilizing the central entry point technique significantly shortened operative times and reduced fluoroscopic exposures.

### 4.2 Anatomical Validation and Gender Discrepancies of Infra‐Acetabular Screw Parameters

Robotic navigation technology has reduced the difficulty of minimally invasive pelvic surgery [10, 15]. However, high‐energy injuries such as acetabular fractures, often accompanied by significant displacement, typically require open reduction followed by internal fixation. Infra‐acetabular screws, as a crucial component of periacetabular screws, provide excellent fixation for acetabular fractures. They facilitate early rigid stabilization, reducing the incidence of postoperative displacement and complications [16, 17] Placement of a 3.5 mm infra‐acetabular screw typically requires a 5 mm bony corridor. In this study, only 78% of male patients (39/50) and 72% of female patients (34/50) had screw corridors ≥ 5 mm. Narrow screw corridors necessitate repeated adjustments and precise placement during infra‐acetabular screw insertion. Therefore, the selection of the entry point is critically important. Baumann analyzed CT images and placed 40 ideal screws, calculating that the average entry point for an ideal infra‐acetabular screw was located 10.2 mm caudal and 10.4 mm medial to the IPE. Furthermore, no significant differences in entry points were observed across different genders, ages, or heights [13]. In this study, no significant differences were observed between males and females regarding entry points. The ideal entry points were located at medial (8.91 ± 2.66) mm and caudal (7.29 ± 2.59) mm for males, and medial (9.42 ± 2.61) mm and caudal (7.49 ± 2.63) mm for females. These coordinates differ from the findings of the Baumann study and are closer to the apex of the iliopubic eminence. However, Lu [11] analyzed 100 Asian pelvises and reported that the ideal entry point in males was located 8.03 mm caudal and 8.49 mm medial to the IPE, while in females it was 8.68 mm and 8.87 mm, respectively. These values are close to the ideal entry point in the present study, suggesting potential racial differences. Western populations have a larger transverse diameter of the pelvic inlet and greater bone channel volume compared to Asian populations [8, 18]. This discrepancy is similarly reflected in the infra‐acetabular screw channel diameter and screw length. Lu [11] reported infra‐acetabular screw corridor diameters of (5.15 ± 1.25) mm and (4.42 ± 1.01) mm for males and females, respectively, which are significantly smaller than the findings in this study: (6.71 ± 0.96) mm and (5.7 ± 0.76) mm. The infra‐acetabular screw corridor diameter in this study was significantly larger than that reported in similar studies [10, 11, 19]. This may be because the present study included only 73 patients with infra‐acetabular screw corridors ≥ 5 mm, resulting in larger corridor diameters. The lengths of the infra‐acetabular screws in males and females were (104.03 ± 3.97) mm and (88.72 ± 3.72) mm, respectively. The male measurement was significantly greater than the female measurement, consistent with other studies [13, 20, 21]. This difference is considered to result from variations in pelvic size and morphology between the sexes.

### 4.3 Morphological Characterization of the Entry Zone and Introduction of the Central Entry Point

The ideal entry point for infra‐acetabular screws is highly concentrated on the medial and caudal aspects of the IPE. In traditional surgery, placement of infra‐acetabular screws relies on the surgeon's experience to adjust the entry point to an area near the ideal location, without a clearly defined entry range. Ma [22] analyzed 106 pelvic models, with entry zone of (314.83 ± 4.79) mm^2^ for males and (198.29 ± 78.70) mm^2^ for females. This differs from the entry zone of the infra‐acetabular screw in males and females in the present study: (422.92 ± 80.31) mm^2^ for males and (195.54 ± 66.57) mm^2^ for females, respectively. Considering the placement of 3.5 mm screws, the actual entry zone requires inward reduction by 1.75 mm. The present study included this portion of the area, resulting in a larger entry zone. The entry zone area in males is significantly larger than in females. The entry zone areas in both groups indicate that the placement of the infra‐acetabular screw offers considerable flexibility in entry point locations, rather than being confined to the ideal entry point. In clinical surgery, the infra‐acetabular screw is typically inserted through the fourth hole of the plate. However, due to variations in the patient's fracture location and plate placement, it is not always possible to position the infra‐acetabular screw at the ideal entry point. Therefore, we investigated all possible entry points for the infra‐acetabular screw and found that the entry zone resembles an elliptical shape. We performed morphological analysis on the entry zone, measuring the major and minor radii of the entry zone. By morphologically matching the entry zone with an ideal ellipse defined by these radii, we found that the SSIM achieved (88.40 ± 5.54) %. Simultaneously, we observed that the coordinates of the ellipse center, like the ideal entry point, exhibit constant height. Therefore, we propose the concept of the central entry point to guide entry point selection. Among the 73 pelvic cases in this study, the central coordinates of the entry point for males and females were: medial to the IPE (10.77 ± 3.19) mm, caudal to the IPE (3.86 ± 1.03) mm; and medial (9.55 ± 2.22) mm and caudal (4.01 ± 1.14) mm for females, respectively. The central entry point consistently lies medial and anterior to the ideal entry point, with no significant gender‐related differences observed. This yields an approximately elliptical infra‐acetabular screw entry zone, with the entry point coordinates maintaining a constant height. During clinical surgery, once the central entry point is identified, even if the plate position is slightly off, the infra‐acetabular screw can be placed more precisely guided by the central entry point and the entry zone.

The 3D morphology of the infra‐acetabular corridor is double‐conical, with the narrowest point being the conical junction. The entry zone for infra‐acetabular screws is similarly constrained by the corridor's narrowest segment [17, 22]. To investigate the diameter of the infra‐acetabular screw corridor, Bastian [23] introduced the radiographic concept of the LCE angle (the angle formed by the perpendicular line from the acetabular center and the lateral acetabular margin). Bastian found that as the LCE angle increases, the distance from the femoral head center to the Teardrop becomes shorter, and the diameter of the infra‐acetabular corridor decreases. Lu [11] measured the minimum thickness of the medial acetabular wall (MTMAW) and the infra‐acetabular corridor diameter (ICAD), finding a linear relationship between the two. Simultaneously, the interspinal diameter may influence the size of the infra‐acetabular corridor by affecting pelvic dimensions. Therefore, we measured the aforementioned parameters and analyzed their correlations with the entry zone area. The results demonstrated that the entry zone area decreased with an increasing LCE angle and increased with increasing interspinous distance and MTMAW, respectively. Measuring these parameters can help surgeons estimate the morphology and dimensions of the entry zone; when combined with the central entry point, these findings may guide a more targeted placement of infra‐acetabular screws.

### 4.4 Clinical Efficacy, Feasibility, and Surgical Efficiency of the Central Entry Point Technique

Based on the aforementioned research, the study team enrolled 20 patients with Anterior and Posterior Column Dissociation fractures of the acetabulum. The following parameters were recorded: screw length, procedure duration, number of radiographs, and screw placement success rate. It was found that there was no statistically significant difference in screw length between the ideal and central entry points for placement of an infra‐acetabular screw. The screw placement success rate was slightly higher in the experimental group than in the control group, but this difference was not statistically significant, possibly due to the small sample size. The experimental group demonstrated significantly shorter screw placement time and fewer x‐ray exposures than the control group, confirming that using the central entry point and the entry zone significantly improves screw placement efficiency.

For a long time, research on infra‐acetabular screws has primarily focused on how to safely insert the screw [12, 24]. Due to the narrow corridor, surgeons must strictly control the entry point and direction of the screw. Deviations may result in the screw cutting through bone or the selected corridor diameter being insufficient for inserting a 3.5 mm screw, leading to an “in‐out‐in” phenomenon. Previous studies have predominantly addressed insertion direction [10, 13, 20], emphasizing adjustment of the insertion angle to facilitate all‐in screw placement. However, clinical practice often lacks precise reference points for fine‐tuning this angle. This study aims to quantify and expand the entry zone, shifting the focus from a single entry point to a broader entry zone, thereby providing additional placement options and enhancing screw fixation for infra‐acetabular screws.

### 4.5 Limitations and Future Perspectives

Limitations of this study: (1) The small sample size and single‐source data may affect statistical power and generalizability. This study included CT data from only 100 patients, all from a single center. The range of variation in the identified entry region may therefore be underestimated. Future multicenter, large‐sample prospective studies are needed to verify and calibrate the parameters proposed in this study. (2) The intraoperative entry point and angle are still highly dependent on the surgeon's experience, lacking objectivity. Although this study defined the ideal central point coordinates through three‐dimensional reconstruction and virtual planning, during actual surgery the precise entry angle and the judgment of the guide pin's contact point on the bone surface remain influenced by the surgeon's tactile sensation and spatial visualization. Subsequent research plans to use the data from this study as a basis for designing patient‐specific positioning guides or developing intraoperative navigation fusion algorithms to reduce reliance on surgical experience. (3) This study does not yet provide a standardized operational guide for rapid intraoperative localization of the central entry point. The current findings are more focused on preoperative planning and have not been translated into simple steps that can be directly used in the operating room (such as surface landmark references or correspondence of bony landmarks under fluoroscopy). This, to a certain extent, limits the clinical translational utility and scalability of the technique.

## 5 Conclusions

The infra‐acetabular screw entry zone is a centrally concentrated, elliptical‐like area, with the ellipse's center located medial and anterior to the ideal entry point. The size of this entry zone correlates with LCE, MTMAW, and Interspinal diameter. Insertion based on the central entry point and entry zone significantly improves screw placement efficiency.

## Author Contributions

**Yongsheng Wang:** data curation. **Jiaqiang Chen:** software, conceptualization, methodology, writing – original draft. **Xiaopan Wang:** formal analysis, validation. **Peishuai Zhao:** software, data curation. **Xiaotian Chen:** validation, formal analysis. **Renjie Li:** data curation, software. **Jianzhong Guan:** funding acquisition, writing – review and editing. **Min Wu:** writing – review and editing, project administration.

## Funding

This work was supported by the Anhui Provincial Department of Education (Grant No. AHWJ2023A10086).

## Disclosure

Authorship Declaration: We hereby declare that all authors listed meet the authorship criteria as defined by the latest guidelines of the International Committee of Medical Journal Editors (ICMJE). All authors have made substantial contributions to the conception, design, data collection, analysis, and/or interpretation of the study, have been involved in drafting or critically revising the manuscript, and have approved the final version for submission. All authors agree to be accountable for the accuracy and integrity of the work.

## Ethics Statement

This study was performed according to the Helsinki declaration and the study was approved by the Medical Ethics Committee of the First Affiliated Hospital of Bengbu Medical University (Number: 2023YJS180).

## Consent

Patients signed informed consent regarding publishing their data and photographs.

## Conflicts of Interest

The authors declare no conflicts of interest.

## Data Availability

The data that support the findings of this study are available on request from the corresponding author. The data are not publicly available due to privacy or ethical restrictions.
